# Analyzing allele specific RNA expression using mixture models

**DOI:** 10.1186/s12864-015-1749-0

**Published:** 2015-08-01

**Authors:** Rong Lu, Ryan M Smith, Michal Seweryn, Danxin Wang, Katherine Hartmann, Amy Webb, Wolfgang Sadee, Grzegorz A Rempala

**Affiliations:** Division of Biostatistics, College of Public Health, The Ohio State University, Columbus, OH 43210 USA; Mathematical Biosciences Institute, The Ohio State University, Columbus, OH 43201 USA; Center for Pharmacogenomics, College of Medicine, The Ohio State University, Columbus, OH 43210 USA; Department of Biomedical Informatics, Program in Pharmacogenomics, College of Medicine, The Ohio State University Wexner Medical Center, Columbus, OH USA

**Keywords:** Allelic RNA expression imbalance (AEI), Allele-specific expression (ASE), RNA-seq, Poisson mixture, Folded Skellam mixture, Human brain

## Abstract

**Background:**

Measuring allele-specific RNA expression provides valuable insights into *cis*-acting genetic and epigenetic regulation of gene expression. Widespread adoption of high-throughput sequencing technologies for studying RNA expression (RNA-Seq) permits measurement of allelic RNA expression imbalance (AEI) at heterozygous single nucleotide polymorphisms (SNPs) across the entire transcriptome, and this approach has become especially popular with the emergence of large databases, such as GTEx. However, the existing binomial-type methods used to model allelic expression from RNA-seq assume a strong negative correlation between reference and variant allele reads, which may not be reasonable biologically.

**Results:**

Here we propose a new strategy for AEI analysis using RNA-seq data. Under the null hypothesis of no AEI, a group of SNPs (possibly across multiple genes) is considered comparable if their respective total sums of the allelic reads are of similar magnitude. Within each group of “comparable” SNPs, we identify SNPs with AEI signal by fitting a mixture of folded Skellam distributions to the absolute values of read differences. By applying this methodology to RNA-Seq data from human autopsy brain tissues, we identified numerous instances of moderate to strong imbalanced allelic RNA expression at heterozygous SNPs. Findings with *SLC1A3* mRNA exhibiting known expression differences are discussed as examples.

**Conclusion:**

The folded Skellam mixture model searches for SNPs with significant difference between reference and variant allele reads (adjusted for different library sizes), using information from a group of “comparable” SNPs across multiple genes. This model is particularly suitable for performing AEI analysis on genes with few heterozygous SNPs available from RNA-seq, and it can fit over-dispersed read counts without specifying the direction of the correlation between reference and variant alleles.

**Electronic supplementary material:**

The online version of this article (doi:10.1186/s12864-015-1749-0) contains supplementary material, which is available to authorized users.

## Background

High-throughput DNA sequencing technology, when used for measuring RNA expression (RNA-Seq), provides nucleotide-level resolution of gene expression across the entire transcriptome in a single experiment. This enhanced resolution provides a wealth of detail about gene expression not available through microarray-based technologies. One important goal is to identify regulatory variants that affect transcription and RNA processing. Use of RNA expression arrays and RNA-Seq to determine transcript levels in multiple samples, combined with single nucleotide polymorphism (SNP) chip genotyping, can reveal expression quantitative trait loci (eQTLs) acting either in *cis* (located at the target gene locus) or in *trans* [1]. A major caveat of eQTLs is their sensitivity to *trans*-acting factors, sometimes making it difficult to attribute changes in expression to a causative variant. On the other hand, allelic mRNA ratios reduce the effect of *trans*-acting factors, revealing the presence of allele-specific regulatory factors acting in *cis* when allelic ratios in the RNA differ from that in gDNA, termed here ‘allelic RNA expression imbalance’ (AEI) [1].

In the literature, the terms AEI or alternatively allele-specific gene expression (ASE) are used to describe the phenomenon when one parental copy of a given autosomal gene is preferentially expressed over the other in the corresponding RNA transcript. Commonly, regulatory variants cause AEI, but epigenetic processes can also be allele-selective, such as with imprinting. Our group and others have taken advantage of the single-base resolution afforded by RNA-Seq to measure allelic RNA expression at heterozygous single nucleotide polymorphisms (SNPs) in the brain [2, 3] and liver [4], among other human tissues [5, 6]. Genomic regions subject to epigenetic programming, such as imprinting, which typically results in large (>10-fold) AEI because of near-complete silencing of one allele, have been identified from RNA-Seq studies of allelic RNA expression in combination with gDNA genotyping [7, 8]. RNA editing can also result in large allelic RNA ratios [2, 3]. Smaller changes in allelic expression can also have biological relevance. However, RNAseq data yield allelic ratios with relatively high noise; therefore, rigorous statistical methods are needed to identify a signature of AEI in transcriptome-wide analyses.

We have repeatedly demonstrated that less extreme AEI ratios resulting from *cis*-acting regulatory variants influence a variety of phenotypes [9], including therapeutic drug response [10, 11], complex genetic disease risk [2, 3, 12, 13], risk for drug dependence [14, 15], cognitive processes [16], and lethal drug overdose [17]. However, current methods for analyzing allelic RNA expression from RNA-Seq have substantial drawbacks when attempting to reliably identify modest allelic differences (<2.5-fold). The main ones are experimental and instrumental noise [18] as well as high read-depth requirements [19]. Even under high-stringency conditions and after grouping allelic ratios from multiple SNPs from the same gene together, our ability to predict modest AEI at low coverage is subject to a considerable false discovery rate [2, 3].

Several methods have been proposed for identifying genes with AEI using RNA-seq data. One class of methods focuses on modeling and correcting for bias involved in generating read counts, such as mapping bias favoring the reference alleles [20–22]. The other class of methods focuses on modeling over-dispersion in read counts, by means of models such as negative-binomial model, Poisson-Gamma model, beta-binomial model, and two-component mixture of beta-binomial model [19, 23–26]. Our method falls into the second class of AEI detection methods and aims to resolve the two problems described in detail below that are difficult to overcome with other existing methods in the same category.

The first problem arises when modeling AEI signals in genes with very few SNPs (<10). To the best of our knowledge, existing models are proposed as single-gene-based methods, with each gene’s reads investigated separately. Based on the rule of thumb (via the cross-validation considerations, see [27]) that estimation of each model parameter requires at least ten observations on average, any single-gene-based model with more than one parameter is only applicable to genes with at least ten heterozygous SNPs, or when data from multiple subjects is available. Taking the human brain dataset analyzed in this paper with RNA-seq (308,912 SNPs called from 98 human brain tissues across ten subjects; SNPs with the same rs number in different brain tissues are counted multiple times), 78 % of genes have 4 SNPs or less in the RNA-seq reads. One can extend the single-gene-based models by aggregating the reads within each gene and applying the models to multiple genes. But in that case, genes with different number of SNPs are treated as directly comparable with each other, ignoring uneven SNP numbers within each gene. Here we use mixture model to group SNPs with similar read coverage across many genes, instead of grouping them by genes. Our approach consists of two modeling stages, one for defining comparable SNP groups and the other for detecting AEI signals within each SNP group.

Another issue with the existing methods for AEI detection is that all the binomial-type models assume a strong negative correlation between reference and variant allele reads. In theory, the RNA expression level of the paternal copy of the gene is independent of the maternal one, but because they are subject to the same cellular environment regulation, the expression levels of the two alleles are likely to be highly positively correlated in the absence of *cis*-acting regulatory variants. Indeed, we observe high correlations between reference and variant read counts in RNA-seq. For instance, in our human autopsy brain tissue dataset discussed below the overall sample correlation between two allele reads is estimated to be 0.92 (cf. Additional file 1: Figure S1). Even after excluding a group of SNPs with the highest read counts, we still see linear correlation around 0.71 between reference and variant reads. The assumption that the reference allele reads follow binomial implies that the theoretical correlation between the reference and variant reads is -1, which is opposite to what is observed in RNA-seq data. The approach taken here is more flexible as it does not assume any specific direction of correlation between reference and variant reads. Note that since our model makes different assumptions than the binomial-type models, it is not easily directly comparable with them via simulation studies.

## Methods

### Postmortem human brain tissues

Human autopsy brain regions were provided from an archived biorepository (University of Miami, Miami, FL, USA), as described in Mash *et al.*, 2007 [28]. Ten subjects (age ranging from 16 to 47 years, five African-American, three European-American, one Pacific Islander, one mixed race) were selected from accidental or cardiac sudden deaths with negative urine screens for illicit drugs, with no history of psychiatric disorders or licit or illicit drug use prior to death; five subjects had a history of cigarette smoking. From each subject, ten different brain regions were obtained: frontopolar cortex (Brodmann Area 10; BA10), Wernicke’s area (BA22), anterior cingulate cortex (BA24), dorsolateral prefrontal cortex (BA46), insular cortex, hippocampus, amygdala, posterior putamen, cerebellum, and brainstem raphe nuclei. In total, our dataset included 98 tissue samples (analysis of two tissues failed). These samples are de-identified prior to attainment.

### Ethics statement

The Office of Responsible Research Practices at The Ohio State University has determined that our study does not meet the federal definition of human subjects research under 45 CFR 46.102(f) [also 32 CFR 219.102(f)]. Therefore, it is waived from further IRB review. This determination is consistent with The Ohio State University Human Research Protection Program (HRPP) policy on human subjects research, found at http://orrp.osu.edu/irb/osupolicies/documents/ResearchInvolvingHumanSubjects.pdf.

### RNA-Seq analysis of transcriptomes and genome-wide SNP genotyping

RNA-Seq transcriptomes were generated from all ten human brain regions in ten different individuals. For each individual, genomic DNA (gDNA) was isolated from the cerebellum and used for genome-wide genotyping with the HumanOmni5Exome BeadChip (Illumina, Inc., San Diego, CA), performed at the University of Utah Genomics Core facility. Total RNA was isolated by homogenizing each tissue in TRIzol, mixing thoroughly with chloroform, and precipitating RNA from the aqueous phase using isopropanol. Total RNA was further purified using SpinSmart™ Total RNA columns (Denville Scientific, Inc, South Plainfield, NJ), and latent genomic DNA (gDNA) was digested on-column with DNase I (QIAGEN Inc., Valencia, CA). Complementary DNA (cDNA) was reverse transcribed from 25 ng total RNA using the Ovation RNA-Seq System v2 (NuGen), which suppresses ribosomal RNA conversion to cDNA and employs both poly-*dT* and random hexamer primers, capturing all RNA species (including non-poly-adenylated RNAs and intronic fragments). This cDNA was used to construct libraries for massively parallel sequencing using the NEBNext DNA Library Prep Set for SOLiD (New England Biolabs, NEB, Ipswich, MA), per manufacturer’s instructions.

Sequenced reads from a 5500 SOLiD System (LifeTechnologies, Menlo Park,CA) (~40 million reads per tissue) were mapped to a modified human genome containing IUPAC ambiguous nucleotide characters for each annotated SNP in dbSNP 135, downloaded from the UCSC Genome Browser, using LifeScope Genome Analysis Software v2.5.1 (Life Technologies, Menlo Park, CA). This method greatly attenuates reference bias alignment, as previously described [2, 3]. Single nucleotide variants were identified with Samtools v0.1.16 [29], which provides a count of the aligned reads containing the reference or variant allele. Identified SNP locations were annotated based on UCSC annotation databases and dbSNP using annovar annotation software [30]. Those polymorphisms confirmed as heterozygous by high-density gDNA genotyping were subsequently included in analyses. Based on annotation, each SNP was assigned to a location within a gene locus—whether exonic, intronic, intergenic, UTR, or upstream/downstream (within 1 kb of the coding region). Exonic, UTR, and intronic counts from coding and non-coding genes were used to calculate allelic RNA expression.

### Folded Skellam mixture model

The Skellam random variable (and the corresponding distribution) is defined as the difference of two independent Poisson random variables [31] and has various applications, for example in image reconstruction [32], financial mathematics [33], and genetics [34]. The term “folded Skellam” refers to the absolute value of the Skellam random variable. In the following model description, we denote the SNP allele reads from the paternal copy of a gene as *P* and that from the maternal copy as *M*. Let *R* and *V* be the reference and variant reads respectively. Although the parental origin of reads is not available in our RNA-seq data, introducing the hidden pair (*P, M*) will help us in justifying the model for analyzing (*R, V*).

One approach to modeling (*P, M*) is to use some discrete bivariate distribution with certain correlation structure. For example, we can assume (*P, M*) follows a mixture of bivariate Poisson distributions. Within each mixture component, the correlation between *P* and (*M*) is modeled by introducing an additive Poisson component, *i.e.*

$$ P={Y}_1+Z,\kern0.5em M={Y}_2+Z $$

where *Y*_1_*, Y*_2_*, Z* follow three independent Poisson random variables. However, the bivariate Poisson mixture model may be not ideal for modeling reads from RNA-seq, as it leads to a restrictive requirement that the marginal distributions have to be univariate Poisson mixtures. In order to be more flexible, in our current approach we only assume that *Y* : = *P* − *M* = *Y*_1_ − *Y*_2_ follows a Skellam mixture distribution with unknown fixed number of mixture components *K*. That is, we make no distribution assumption on the shared additive component Z. Consequently, the joined density of (*P, M*) is

$$ {f}_{P,M}\left(p,\kern0.5em m\Big|\pi, \kern0.5em \varLambda \right):={\displaystyle {\sum}_{i=1}^K\left({\displaystyle {\sum}_{z=0}^{\min \left(p,\kern0.5em m\right)}{\pi}_i\mathrm{Poisson}\left(p-z\Big|{\lambda}_{i,p}\right)\mathrm{Poisson}\left(m-z\Big|{\lambda}_{i,m}\right){f}_{z_i}(z)}\right)} $$

where $\pi =\left({\pi}_1,\dots, {\pi}_K\right),\varLambda =\left(\left({}_{\lambda_{1,m}}^{\lambda_{1,p}}\right),\dots, \left({}_{\lambda_{K,m}}^{\lambda_{K,p}}\right)\right)$ are the model parameters and ${\left\{{f}_{Z_i}(Z)\right\}}_{i=1}^K$ is a set of unknown probability mass functions.

Since we expect to have |*R* − *V*| = |*P* − *M*| it follows that |*R* − *V*| should have the same folded Skellam mixture distribution as |*P* − *M*| in our setting. Since the mean of the Skellam variable equals the difference of two corresponding Poisson means, testing the null hypothesis of no AEI signal within a mixture component is equivalent to testing whether the means of two independent Poisson variables are equal. That is, if the component *i* is a “no AEI signal” component, then under our model λ_*i*,*p*_ = λ_*i*,*m*_ = : λ and we can estimate λ by the method of moments using the fact that *E*(*R* − *V*)^2^ = *E*(|*R* − *V*|)^2^ = 2λ.

### Mixture model pipeline for AEI signal detection

AEI is often measured using the ratio of reads aligned to the reference and the variant allele. The ratios in RNA from autosomal genes observed to deviate significantly from unity are considered as AEI signals. The reliability of many currently applied AEI measures depends on the stringency of the threshold for assigning AEI, and we have previously used allelic differences of 1.5-fold or greater to assign possible AEI [2, 3]. However such arbitrary threshold may not be very efficient in optimizing the missed and false discovery rates for AEI calls. Since the Skellam mixture model described above takes advantage of read counts information across all genes, including those with small number of SNPs (<10), it is expected to have better ability to detect AEI.

Under the null hypothesis of no AEI signal, we assume that the fluctuations in sequence read differences (between reference and variant alleles) across multiple SNPs are comparable with each other when the sequencing coverage (*i.e.*, the sum of reference and variant allele reads) is of similar magnitude across these SNPs. We refer to such SNPs as “comparable”. Accordingly, we first categorize the comparable SNPs based on the sequencing coverage counts (rescaled after library size adjustments) using a finite mixture of univariate Poisson distributions, and subsequently search for AEI signals within each group of comparable SNPs by fitting a folded Skellam mixture model to the absolute values of rescaled read differences. This approach provides an alternative way of making AEI signal calls in a manner which is more reflective of the noise structure in the RNA-seq data and thus enables considerations of AEI under improved signal to noise ratio, without overly restrictive *a priori* fold-change thresholds like 1.5, *etc.*

Although in most genetic applications one does prefer to represent AEI as a read count ratio rather than a read count difference, under our additive interaction model between *P* and *M* there is a clear advantage in considering the latter along with the former. To compensate for the relatively noisy raw read counts differences, we propose to include library-size adjustments of the originally observed read pairs (the reads of reference and variant alleles at the same locus are considered a pair) while preserving the ratios of the raw counts, and group “comparable” SNPs before modeling the differences of adjusted read counts. The major advantage of using discrete distributions like Poisson and Skellam in our modeling is that we can fit low counts data well, unlike most smoothing techniques and Gaussian-type approximations. This is important, since, for instance, in our human brain dataset 95 % of all 10,702 pairs of read counts at identified SNP sites are low counts (<33 reads) (summary statistics are provided in supporting information Additional file 2: Table S1). Below we describe the Skellam-based pipeline for detecting AEI signals in the brain whole transcriptome sequencing datasets.

Step 1: Library size adjustment

To account for differences in the depth at which each tissue sample was sequenced, we multiply each pair of read counts by the ratio of the median total number of reads across all tissue samples to the total number of reads for the specific sample from which the reads are generated. The scatter plots of read pairs, with and without library size adjustment, are presented in supporting information section Additional file 1: Figure S1. Note that adjusting for the library sizes does not alter the ratio between two reads in the original dataset.

Step 2: Classifying the sum of read counts

To facilitate AEI signal detection in read pairs with different magnitudes, we first group SNPs according to the sequencing coverage. By treating each gene from subject-specific brain tissue as a unit, we first average the sum of adjusted reads within each unit, and then fit a finite Poisson mixture model to those reads-sum averages. We use the Expectation-Maximization (EM) algorithm for fitting the Poisson mixture [35], and use Bayesian information criterion (BIC) to set the optimal number of mixture components (*i.e.* the number of SNP groups). Based on the fitted model (Table 1), each of the subject-and-brain-region-specific gene units can be classified into the Poisson mixture components. Therefore, for instance, genes with very few SNPs are grouped with other genes with similar number of averaged total reads.

**Table 1:** Poisson mixture model parameter estimates and SNPs classification results

Mixture component	Proportion	Poisson mean	No. of SNPs	No. of genes
Comp.1	*0.030 (0.029, 0.031)*	*43.11 (42.54, 43.84)*	*18367*	*784*
Comp.2	0.0011 (0.0010, 0.0012)	152.37 (146.08, 166.13)	519	37
Comp.3	0.186 (0.182, 0.190)	20.34 (20.20, 20.49)	82963	3892
Comp.4	0.003 (0.0025, 0.0033)	108.14 (105.13, 115.60)	2073	89
Comp.5	0.0006 (0.0004, 0.0008)	201.01 (196.15, 209.71)	425	27
Comp.6	0.0073 (0.0069, 0.0077)	74.60 (72.56, 78.08)	5156	202
Comp.7	0.771 (0.769, 0.775)	7.82 (7.78, 7.85)	198889	11174

The Poisson mixture model was fitted to the averaged total reads within tissue-specific genes (62326 tissue-specific genes in total, *i.e.* sample size = 62326; overall log-likelihood = -216846; BIC = 433836). Genes with the same rs number but from different brain region were considered as different tissue-specific genes. We found the optimal number of mixture components to be 7, meaning that we could classify all SNPs into 7 “comparable” SNP groups. Most SNPs in the gene of our interest (*SLC1A3*) were classified into the mixture component Comp.1. The SNPs in Comp.1 were used to fit the folded Skellam mixture model

Step 3: Classifying the differences of read counts

Before analyzing count differences between variant and reference reads, we further divide the set of count pairs within each Poisson mixture component into another four smaller subsets of read pairs according to their location within a gene: 3’ UTR, 5’ UTR, intron, or exon. This step of the algorithm accounts for the fact that the read count differences or ratios from different genetic regions can differ in magnitude. For example, introns are expected to have lower expression than exons. Furthermore, read ratio differences between these regions can occur due to RNA isoforms generated by alternative splicing or different UTR usage at a given gene locus. Accordingly, further statistical analyses are done separately within each subpopulation. For example, we can first evaluate the subset of all adjusted count pairs that are classified into the first Poisson mixture component and also labeled as reads from the 3’ UTR. We use mixture of folded Skellam distributions to model absolute values of these rescaled read differences and classify data into separate folded Skellam components. For fitting the folded Skellam, we used a likelihood-free Markov chain Monte Carlo (MCMC) method [36], which can be also viewed as an Approximate Bayesian Computation (ABC) type of method [37].

Step 4: Testing for signal significance

We define AEI signals as the count pairs being classified into folded Skellam mixture component with significantly different Poisson means. A likelihood ratio testing (LRT) procedure is used for assessing significant differences in the two parameters of a folded Skellam distribution. Given the subset of count pairs classified into one folded Skellam mixture component, the folded Skellam parameter (equal Poisson means) under the null hypothesis can be estimated using the method of moments (see the previous section on folded Skellam mixture model), and then the log-likelihood of observing such set of differences under the null hypothesis can be calculated accordingly. To evaluate the log-likelihood without the null hypothesis constraints, we used the corresponding parameter estimates obtained in the process of fitting the overall folded Skellam mixture model. The LRT statistics are compared to a chi-square distribution with one degree of freedom.

## Results and discussion

To present the potential of decomposing signals from RNA-seq data using the mixture model pipeline, we consider the dataset described above in which we focus only on pairs of counts with at least 3 reads for the allele with lower expression (min(*R*, *V*) ≥ 3) and exclude intergenic SNPs.

### Poisson mixture model fitting results

After normalizing the RNA-seq dataset (see pipeline step 1), we fit the Poisson mixture model and find the optimal number of seven components using the BIC criterion. We note that since the Poisson mixture model is expected to reflect the experiment-specific RNA-seq frequency patterns, the particular number of components does not seem to have any meaningful (biological) interpretation. Overall, as long as the mixture model reasonably well fits the data, our downstream analysis is expected to be robust with respect to the number of components. For practical reasons, we remove the 0.1 percent of the highest average of scaled counts over different gene by tissue categories. Table 1 presents the results of this fitting procedure. We note that over 90 % of the genes are contained in mixture components Comp.3 and Comp.7. Accordingly, we expect these two components to contain most of the genome-wide signal. In order to compare our final AEI predictions against those previously reported in the literature in the same dataset [2, 3], we limit ourselves only to the variants in genes from the first Poisson mixture component (Comp.1) and select the genetic location with the highest number of heterozygous positions aligned, namely the 3’UTR, as noted in Table 2. In many genes, read counts are greatest in the 3’-UTR because of the use of poly-*dT* primes in addition to random hexamers, facilitating detection of AEI in the 3’-UTR.

**Table 2:** Poisson mixture Comp.1 SNP counts by gene regions

	3’ UTR	Exon	Intron	5’ UTR
**No. of SNPs**	**10702**	4694	2142	269
**No. of Genes**	**531**	405	236	43

In total 18367 SNPs were classified into the Poisson mixture component 1 and 10702 of them were in 3’ UTR of 531 genes. Fitting of the folded Skellam mixture model only used the 10702 SNPs in 3’ UTR

### Folded Skellam mixture fitting results

**Fig. 1:**
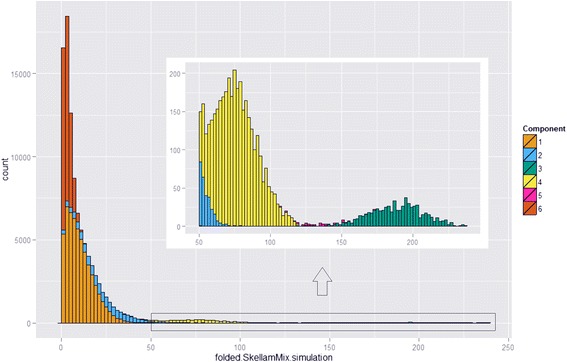
Histogram of the simulation from the folded Skellam mixture (sample size = 10^5^). Different mixture components are indicated by different colors. The two mixture components Mix1 and Mix6 which are closest to zero are considered the two no AEI signal components. The right tail (>50) with relatively smaller frequencies is enlarged and presented in the inner panel

We fit the folded Skellam mixture model to the adjusted read pairs classified into the first Poisson mixture component, and only use SNPs on the 3’ UTR. After performing classification of these SNPs, we identify two AEI signal components (Mix2 and Mix4) and two no AEI signal components (Mix1 and Mix6) (Table 3) by using the LRT (see pipeline step 4). To help visualize the fitted mixture model, we simulated 10^5^ counts from the fitted folded Skellam mixture where we represented different mixture components with different colors (Fig. 1). The histograms of the observed absolute read differences indicating classification to the mixture components are available in supporting information Additional file 3: Figure S2. The goodness-of-fit analysis for the mixture model was performed by plotting the percentiles of absolute read differences against those of counts simulated from the fitted model. Since the absolute read differences from 10,702 SNPs have a long and sparse tail on the right-hand side (95^th^ percentile is 29 while the maximum is 221), we expect the fit in the tail to be relatively poor. Note that this should not, however, adversely affect the quality of the AEI calls since the large values are most likely to be classified as AEI SNPs anyway. In the context of screening for AEI signal, the key to fitting the folded Skellam mixture is to get accurate fit on data points that are close to zero (*i.e.*, to identify the smallest AEI signal component). Based on the Q-Q plots (Additional file 4: Figure S3 in supporting information) we conclude that the fitting is reasonably good up to the 94^th^ percentile of the data.

**Table 3:** Folded Skellam mixture parameter estimates and results of LRTs for equal Poisson mean values

Parameter	Mix1	Mix2	Mix3	Mix4	Mix5	Mix6
*π*_*i*_	0.54 (0.54, 0.55)	0.1 (0.10, 0.11)	0.0065 (0.0064, 0.0066)	0.037 (0.036, 0.038)	0.0003 (0.0003, 0.00035)	0.3 (0.3, 0.31)
*λ*_*i*,1_	65.7 (65.4, 66.5)	83.8 (82.6, 84.2)	268 (263.3, 269.4)	92.7 (91.4, 93.1)	214.8 (212.2, 216.3)	4.81 (4.75, 4.84)
*λ*_*i*,2_	69.2 (69.2, 70.2)	106 (105, 107)	80.3 (79.9, 81.5)	166.0 (165.9, 169.1)	78.1 (77.0, 78.5)	5.39 (5.29, 5.40)
***L***_**0**_	−17852	−2074	NA	−650	NA	−7860
***L***_**1**_	−17864	−1967		−522		−8233
***p*** **-value**	1	<0.00001		<0.00001		1
**No. of SNPs**	5459	482	3	130	2	4626
**No. of Genes**	471	165	3	72	2	407

Only SNPs on 3’ UTR and classified into Poisson mixture component 1 were used for fitting the folded Skellam mixture (overall log-likelihood = -34979; BIC = 70117; sample-size = 10702; (*λ*_*i*,1_, *λ*_*i*,2_) is estimate of the ordered pair (*λ*_*i*,*P*_, *λ*_*i*,*M*_). NAs indicate insufficient sample sizes for LRTs

We do not use LRTs for mixture component Mix3 and Mix5 because there are too few SNPs (5 SNPs in total) being classified into these two components. However, since both Mix3 and Mix5 are even further away from zero than Mix2, which is already designated as the AEI signal component by LRT, it is reasonable to call Mix3 and Mix5 the AEI signal components as well. Accordingly, we consider 5 SNPs in Mix3 and Mix5 as AEI signal SNPs. Additional file 5: Table S2 (see supporting information) lists the raw read counts of these 5 SNPs, along with the mixture probabilities of these 5 SNPs belonging to each of the six folded Skellam distributions, all with relatively high read coverage and absolute ratio of read counts above 2. The mixture probabilities of these 5 SNPs belonging to Mix1 or Mix6 (the two no AEI signal components) are all zero, indicating the significant AEI signals.

Overall, since the two no AEI mixture components contain about 84 % of the data, we conclude that the remaining 16 % of tested SNPs (1,712 out of 10,702) appear to carry statistically significant AEI signals under the model assumptions. However, by classifying SNPs into folded Skellam mixture components according to the largest mixture probabilities, we only identified 617 AEI signal SNPs out of the total 10,702 “comparable” SNPs, indicating that only about 6 % of tested SNPs can be designated as AEI signal with the classification done according to the maximum value of the six mixture probabilities. The remaining 10 % cannot be considered as statistically significant AEI signal sources, although according to our model they did display some evidence of AEI.

### Model performance analysis

To understand better the characteristics of AEI SNPs that stand out in the screening of our mixture model pipeline, and to investigate the relationship between mixture model pipeline and the commonly employed allele ratio threshold, we first tabulate separately the percentiles of absolute read ratios (*i.e.* Max(*R,V*)/Min(*R,V*)) for the 617 AEI SNPs and all remaining 10,085 SNPs (in Mix1 and Mix6, mix of 10 % uncertain AEI signal SNPs and no AEI signal SNPs) (Table 4). Approximately 90 % of these 617 AEI SNPs have absolute read ratios above 1.54, while 60 % of the 10,085 mixture SNPs have absolute read ratios below 1.54. Since 10,085 mixture SNPs contain approximately 10 % uncertain AEI signal SNPs (1,712-617 = 1,095 uncertain AEI SNPs), high absolute read ratios (>2.5) are also expected in the 10,085 SNPs mixture.

**Table 4:** Percentiles of absolute read ratios

SNP category	Min	10 %	20 %	30 %	40 %	50 %	60 %	70 %	80 %	90 %	Max
**617 AEI signal SNPs**	1.14	**1.54**	1.71	1.88	2.08	2.32	**2.64**	3.06	3.67	4.85	9
**10,085 SNPs mixture**	1	1.05	1.13	1.2	1.29	1.4	**1.54**	1.71	2	**2.5**	9.67

Absolute read ratios were calculated using the formula Max(reference, variant)/Min(reference, variant). The 617 AEI signal SNPs were designated according to the largest mixture probability. The remaining 10,085 SNPs included 10 % uncertain AEI signal SNPs and 84 % no AEI signal SNPs

To investigate further the behavior of our mixture model based AEI detection pipeline, we additionally analyze SNPs designated as having AEI despite a low ratio between the alleles and those designated as not having AEI despite a high ratio between the alleles. Among the 617 AEI signal SNPs, there are 51 SNPs with absolute read ratios less than or equal to 1.5 and 9 with absolute read ratios less than or equal to 1.3. In the 10,085 SNPs mixture, 1,003 SNPs have absolute allelic ratio above 2.5, while 10 have absolute read ratios above 7. Detail information of the 9 AEI signal SNPs with the smallest ratio values and the 10 uncertain mixture SNPs with the largest ratio values are listed in Additional file 6: Table S3 and Additional file 7: Table S4 (see supporting information), respectively. None of the 9 AEI signal SNPs has more than 75 % aggregated probability of being in the signal components (Mix2 through Mix5). If the mixture component classifications were done using 80 % probability being in signal components as the criterion, none of the 9 SNPs would be classified as AEI signal SNP. Obviously, the higher required confidence level, the fewer AEI signal SNPs can be identified. For the uncertain mixture SNPs in Additional file 7: Table S4, the main reason for SNPs with very high read ratios failing our pipeline screening is that the raw read counts are too low. The minimum values of these SNP read pairs are either exactly three (threshold for calling a SNPs) or only one or two reads higher. Additionally, some of these small read differences have even smaller library-size-adjusted differences because the corresponding library sizes are above the median level. On the other hand, there are 143 SNPs (supporting information Additional file 8: Table S5) out of the total 617 AEI signal SNPs (supporting information Additional file 9: Table S6) that have more than 99 % probability of carrying AEI signals under the folded Skellam mixture model. For these 143 99 % confident AEI signal SNPs, the mean (median) raw reads of reference and variant alleles are 120 (105) and 75 (31) respectively, while the mean (median) read ratio is around 3.36 (3.21). Therefore, in general, SNPs need both high reads ratio and high reads coverage to pass our mixture model based for robust AEI signals.

### SNP-level AEI signals on gene *SLC1A3*

Smith *et al.* (2013b) [3] previously characterized allelic RNA expression using nine brain regions from a single sample from the same dataset (MB011), finding large and consistent allelic differences for multiple genes, including *SLC1A3*. AEI in this gene was confirmed using a targeted PCR-based SNaPshot method to measure allelic RNA ratios [3]. Our mixture model pipeline classifies ten subject-and-tissue-specific SNPs on this gene into AEI signal components. Within subject MB059, SNP rs2269272 in *SLC1A3* is identified twice as being (with 99 % confidence) AEI signal SNP in two brain regions, insula and amygdala. Within subject MB052, the same SNP (rs2269272) is again identified as AEI SNP with relatively less confidence, but in the same two brain regions (insula and amygdala). Additionally, SNPs rs1049524, rs104922 and rs10428531 in *SLC1A3* are also classified as AEI signal SNPs in one or more brain regions in different subjects including MB011, consistent with previous results [3]. Together, these findings argue for the presence of at least one *cis*-acting regulatory genetic variant that changes expression of *SLC1A3* mRNA.

### Signal designation consistency across brain tissues

Generally speaking, within the same subject, when one SNP locus in one brain region is showing AEI we expect to see the same SNP locus showing AEI signals consistently across most of the other brain regions, unless the regulatory effects are tissue or brain region selective. Using the maximum mixture probability as the criterion, we can compare the number of times that a specific SNP locus is identified as AEI signal across multiple brain regions with the total number of times it is expressed within the same subject. By including only SNPs with read coverage observed in at least two brain regions from the same subjects, we find that there are 114 subject-specific SNPs showing AEI signals in at least half of the brain regions where we have observed expressions. Among these 114 SNPs, over 50 % SNPs show consistent AEI signals in more than one region, while some show consistent AEI signals in all regions that the gene expresses. For example, *SLC24A2* SNP rs7872265 expresses in five brain regions (brain region BA10, BA22, BA24, raphaenucleus, and BA46) and shows AEI in all five regions in MB011. Any inconsistent results in different brain regions may be caused by relative low count coverage in one or more regions and/or lower AEI ratios. We also cannot rule out the possibility of different splice variants or 3’UTR usage in different brains regions, which can confound AEI analysis.

### Comparison between the results from mixture model and whole gene filtering method

An alternative analysis for the AEI detection known as the whole gene filtering method (described fully in Smith *et al.*, 2013b [3]) was carried out on the same brain tissue samples analyzed above, with some additional replicate sequencing runs. The main differences between the two methods are summarized as follows: 1. The mixture model pipeline scans for AEI signals at the SNP level, while the whole gene filtering method scans for AEI signals at the gene level; 2. For the whole gene filtering method, the read ratios of SNPs in all genetic regions (3’ UTR, exon, intron, and 5’ UTR, *etc.*) on the same gene are averaged to get a gene-level expression imbalance measurement, while fluctuations in SNPs from different genetic regions are considered non-comparable in the mixture model and modeled separately.

3. SNPs are not called in the whole gene filtering method if the corresponding genes have only one SNP expressed, while these SNPs are still used and classified in the mixture model pipeline as long as both the reference and variant allele read counts are above 3 (the predetermined threshold). Overall in our comparisons the mixture model appears to be more sensitive to identifying AEI signal than the whole gene filtering method, yielding more AEI signal SNPs. For example, the 592 SNPs identified by the mixture model pipeline with AEI were not identified by the alternative method, likely because their limited coverage or SNP calls across the gene. These 592 instances include 287 unique SNPs present in 175 genes. On the other hand, 90 SNPs identified by the whole gene filtering method failed to be detected in the mixture model pipeline. Interestingly, 84 % of these were assigned into the first folded Skellam mixture component (Mix1) indicating that there was a notable difference between allele counts, but not enough evidence for the final AEI designation, possibly caused by low coverage or low AEI signal as discussed above. Since the mixture model method used only SNPs in 3’UTR, while the genome filter method used all SNPs along the expressed gene locus (from 5’ to 3’UTR), the discrepancy could also be caused by different 3’UTR usage or overlapping neighboring genes.

### Parallels between AEI and eQTLs

The goal of AEI analysis is to identify functional regulatory variants, which are speculated to underline many association signals in genome-wide association studies or eQTL analyses. We have used the Genotype-Tissue Expression Project (GTEx) data to test for the potential of the AEI signal SNPs to reveal the presence of eQTLs. The eQTLs were extracted from transcript counts over all tissues and individuals available in the first release of the GTEx data (56 tissues; 216 individuals). We have normalized the transcript read counts using the function ‘estimateSizeFactors’ in the Bioconductor package ‘DESeq’ (http://bioconductor.org/packages/release/bioc/html/DESeq.html), and to make our analysis more robust to low counts, we have summed all transcript reads in a given gene, obtaining a single expression value for each gene across all tissues. Next, we have stratified individuals by genotype (homozygous major, heterozygous, and homozygous minor) for each SNP with available genotype data (genotyping was performed on Illumina 5 M and Illumina exome chips) - here we did not use imputation to avoid losing statistical power. Finally, we used standard linear regression to test whether the expression level is dependent on the genotype. Of AEI SNPs (in components Mix2 and Mix4) that were directly genotyped 17.6 % (18) reached the standard statistical level of significance (0.05) in the linear regression model (supporting information Additional file 10: Table S7). Of SNPs without evidence for AEI (in component Mix6), a much lower percentage, 9 % (37), were statistically significant eQTLs. Using the ‘sm’ package in R (http://www.r-project.org), we compared the distributions of p-values for association with gene expression between AEI and no AEI SNPs. Overall we observed a non-significant trend of lower p-values among AEI SNPs.

## Conclusion

This study provides a novel framework to determine cases of AEI, and hence *cis-*acting regulatory factors, from RNA-seq data. The method is particularly useful when scanning for AEI signals in RNA-seq datasets having a large number of genes with small number of heterozygous SNPs (<10) from multiple tissues. Our method ensures that all read counts get analyzed simultaneously and all contribute to the AEI classification for each SNP. It also utilizes both the sum and the difference of the adjusted read counts while preserving the raw count ratios throughout the entire analysis. For instance, the mixture model we propose treats a pair of reads (1, 2) differently from (100, 200), while they are viewed exactly the same by ratio statistics. As a consequence, our method can also detect AEI signal that is below the commonly used ratio threshold as long as the signal is consistent and robust, in the sense that there is a sufficient number of large read differences. The robust threshold values typically applied for AEI calling using gene-based criteria seem to result in poor overlap between AEI calls based on the folded Skellam mixture and the ratio threshold approach. However, as long as its model assumptions are valid, our mixture method can make corrections in AEI calls once more data or information becomes available, which is not the case for the predetermined thresholds where the accuracy of AEI classification criterion cannot be improved regardless how much additional data is collected. Finally*,* unlike the binomial-type Bayesian models, ours does not assume (or require) a strong negative correlation between reference and variant allele reads. Some drawbacks of using mixture models need to be pointed out as well. Because of the identifiability issues [38], fitting of a mixture model is often computationally challenging and expensive, and the confidence intervals obtained by MCMC or ABC type methods may be sometimes too wide for meaningful interpretation with small amount of reads. Since our mixture model provides an unsupervised AEI detection method, it is sensitive to the underlying parametric assumptions.

By applying the folded Skellam mixture model to RNA-Seq data from human autopsy brain tissues, we find that within a group of 531 “comparable” genes, 16 % SNPs in the 3’UTR show AEI, which compares favorably with other similar studies. For instances, Dimas *et al.* analyzed allelic expression in different HapMap populations, including 60 Caucasians, 45 Chinese, 45 Japanese, and 60 Yoruba, and found approximately 18 % human genes show AEI [39]. Serre *et al.* performed AEI analysis on more than 80 individuals of European descent for 2,968 SNPs located in 1,380 genes, and found about 20 % human genes show AEI [40]. Most recently, Zhang *et al.* proposed a two component beta-binomial mixture for AEI analysis, and they concluded that approximately 17 % genes within a single individual show AEI [24]. Our present findings seem to be consistent with these results.

### Availability of supporting data

The data set supporting the results of this article is available in the GEO repository (accession ID: GSE68559; http://www.ncbi.nlm.nih.gov/geo/query/acc.cgi?token=kvaxwogwdxgjvwx&acc=GSE68559).

## Additional files

Additional file 1: Figure S1. Scatter plots of reference allele reads versus variant allele reads. The two panels on left hand side are the reads distributions before library size adjustments, and the two panels on the right are the reads distributions after library size adjustments. Additional file 2: Table S1. Summary statistics of reference and variant allele read counts. The total number of SNPs is 308,912. Additional file 3: Figure S2. SNPs classification results based on the fitted folded Skellam mixture. In the histogram of absolute value of adjusted read differences, folded Skellam mixture classification results are indicated by colors. The upper panel shows the overall range of all absolute value of adjusted read counts differences with bin width 2. Two lower panels divide the domain of the histogram in the upper panel at 50, and show the distributions separately to facilitate visualization of the right tail. The bin width in lower left panel is 2 and that in lower right panel is 10. Additional file 4: Figure S3. Folded Skellam mixture model goodness-of-fit plots. Panel 1 is the histogram of absolute values of adjusted differences between reference and variant allele read counts (variable name is “y”), with bar width = 1. Panel 2 is the histogram of simulated counts using the fitted folded Skellam mixture model (variable name is “y.s”), with bar width = 1. Panel 3 shows the scatter plot of “y” percentiles versus “y.s” percentiles, with 2 % data between every two successive circle points. Panel 4 shows the scatter plot of “y” percentiles versus “y.s” percentiles up to their 94^th^ percentiles, with 1 % data between every two successive circle points. Additional file 5: Table S2. SNPs classified in folded Skellam mixture component Mix3 and Mix5. “ref” and “var” are the original read counts of reference and variant alleles without the adjustment for library sizes. Abs.Ratio = Max(ref, var) / Min(ref, var). “Abs.Adj.Dif” is the absolute value of read difference between reference and variant alleles after library size adjustments. {P*i*}, *i* = 1, 2, … 6, are the mixture probabilities corresponding to each of the six folded Skellam mixture components. Only SNPs in 3’ UTR were used for fitting folded Skellam mixture. Additional file 6: Table S3. AEI signal SNPs with absolute read ratios less than or equal to 1.3. “ref” and “var” are the original read counts of reference and variant alleles without the adjustment for library sizes. Abs.Ratio = Max(ref, var) / Min(ref, var). “Abs.Adj.Dif” is the absolute value of read difference between reference and variant alleles after library size adjustments. {P*i*}, *i* = 1, 2, … 6, are the mixture probabilities corresponding to each of the six folded Skellam mixture components. “Comp.” is the assigned folded Skellam mixture component. Additional file 7: Table S4. Uncertain Signal SNPs with absolute read ratios greater than or equal to 7. “ref” and “var” are the original read counts of reference and variant alleles without the adjustment for library sizes. Abs.Ratio = Max(ref, var) / Min(ref, var). “Abs.Adj.Dif” is the absolute value of read difference between reference and variant alleles after library size adjustments. {P*i*}, *i* = 1, 2, … 6, are the mixture probabilities corresponding to each of the six folded Skellam mixture components. “Comp.” is the assigned folded Skellam mixture component. Additional file 8: Table S5. AEI signal SNPs designated with 99 % confidence (n = 143). Brain region 1 through 10 refer to brain region BA10, BA22, BA24, insula, amygdala, hippocampus, postputamen, cerebellum, raphaenucleus, BA46 respectively. “ref” and “var” are the original read counts of reference and variant alleles without the adjustment for library sizes. Abs.Ratio = Max(ref, var) / Min(ref, var). {P*i*}, *i* = 1, 2, …, 6, are the mixture probabilities corresponding to each of the six folded Skellam mixture components. “Comp.” is the assigned folded Skellam mixture component. Additional file 9: Table S6. AEI signal SNPs designated using the maximum mixture probability (n = 617). Brain region 1 through 10 refer to brain region BA10, BA22, BA24, insula, amygdala, hippocampus, postputamen, cerebellum, raphaenucleus, BA46 respectively. “ref” and “var” are the original read counts of reference and variant alleles without the adjustment for library sizes. Abs.Ratio = Max(ref, var) / Min(ref, var). {P*i*}, *i* = 1, 2, …, 6, are the mixture probabilities corresponding to each of the six folded Skellam mixture components. “Comp.” is the assigned folded Skellam mixture component. Additional file 10: Table S7. SNPs classified as AEI signal SNPs and the corresponding gene classified as eQTLs (n = 18). *P*-values are calculated using linear regression.
