# Supplementary figures and images for: Analyzing allele specific RNA expression using mixture models
Source: BMC Genomics. 2015 Aug 1;16(1):566. doi: 10.1186/s12864-015-1749-0 (PMC4521363; doi:10.1186/s12864-015-1749-0)

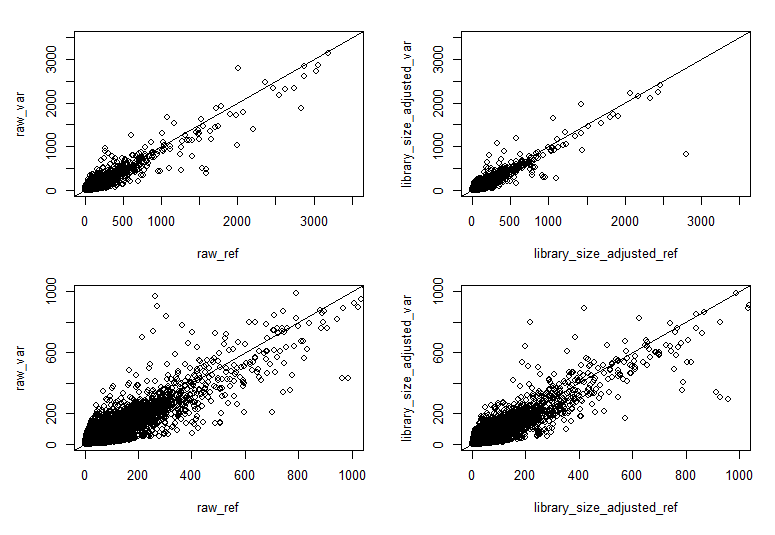

Supplement: Additional file 1: Figure S1. — Scatter plots of reference allele reads versus variant allele reads. The two panels on left hand side are the reads distributions before library size adjustments, and the two panels on the right are the reads distributions after library size adjustments. [file 12864_2015_1749_MOESM1_ESM.png]

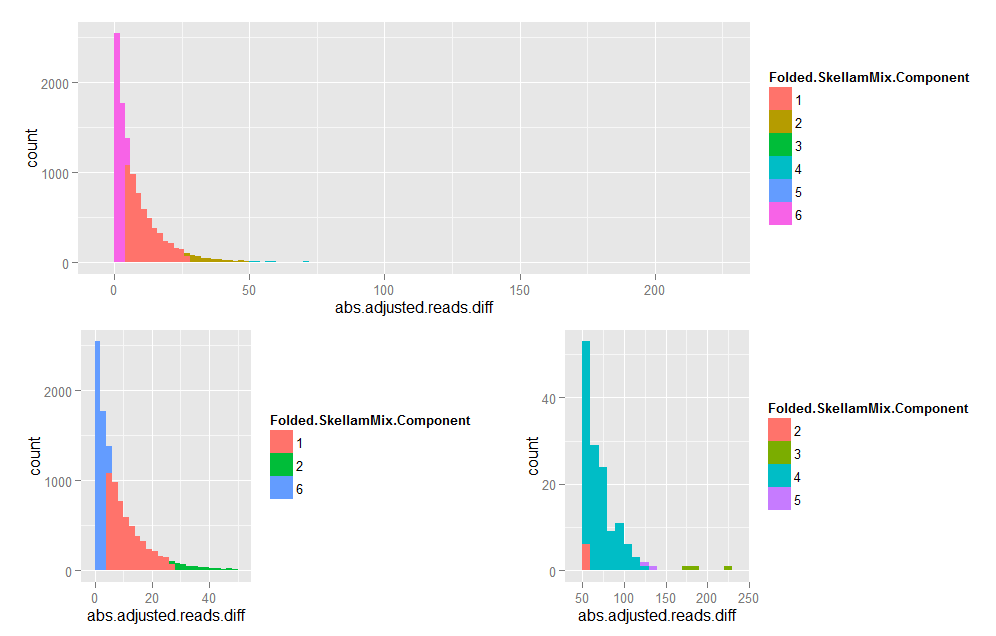

Supplement: Additional file 3: Figure S2. — SNPs classification results based on the fitted folded Skellam mixture. In the histogram of absolute value of adjusted read differences, folded Skellam mixture classification results are indicated by colors. The upper panel shows the overall range of all absolute value of adjusted read counts differences with bin width 2. Two lower panels divide the domain of the histogram in the upper panel at 50, and show the distributions separately to facilitate visualization of the right tail. The bin width in lower left panel is 2 and that in lower right panel is 10. [file 12864_2015_1749_MOESM3_ESM.png]

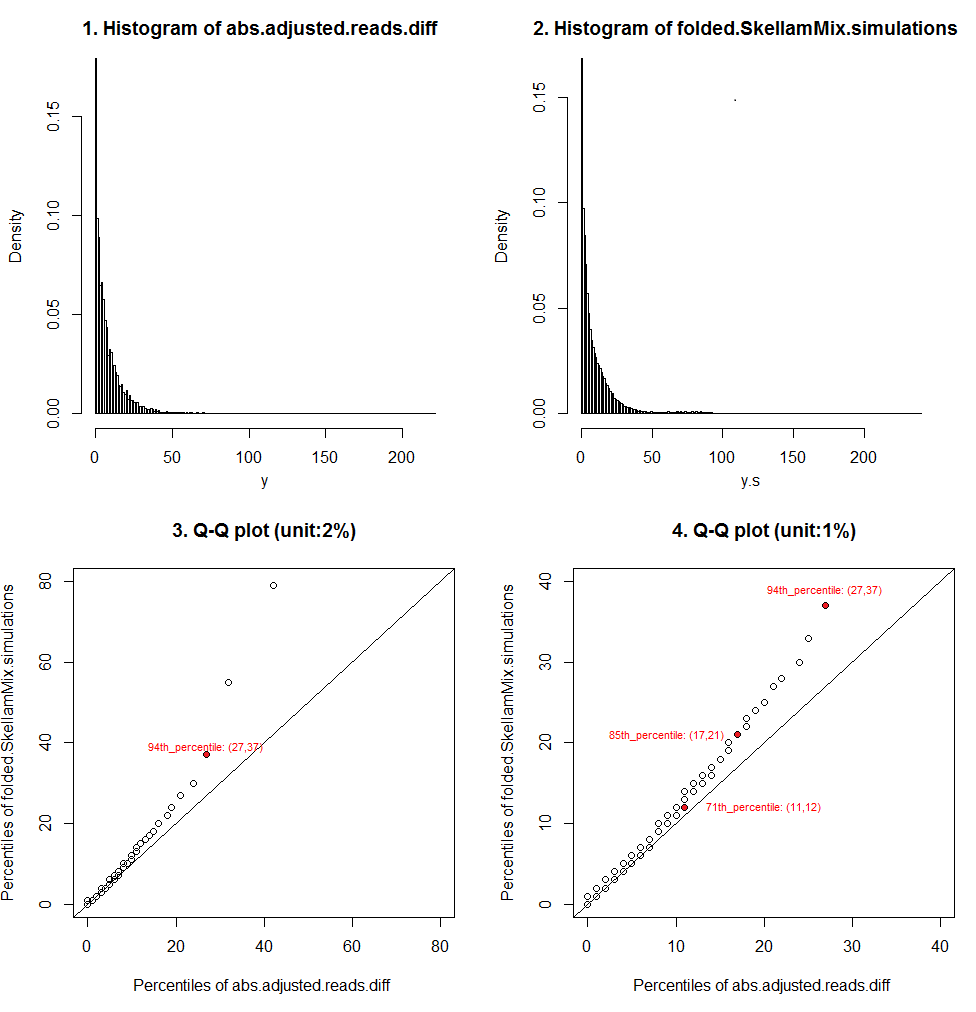

Supplement: Additional file 4: Figure S3. — Folded Skellam mixture model goodness-of-fit plots. Panel 1 is the histogram of absolute values of adjusted differences between reference and variant allele read counts (variable name is “y”), with bar width = 1. Panel 2 is the histogram of simulated counts using the fitted folded Skellam mixture model (variable name is “y.s”), with bar width = 1. Panel 3 shows the scatter plot of “y” percentiles versus “y.s” percentiles, with 2 % data between every two successive circle points. Panel 4 shows the scatter plot of “y” percentiles versus “y.s” percentiles up to their 94th percentiles, with 1 % data between every two successive circle points. [file 12864_2015_1749_MOESM4_ESM.png]
