# Supplementary material for: Analyzing allele specific RNA expression using mixture models
Source: BMC Genomics. 2015 Aug 1;16(1):566. doi: 10.1186/s12864-015-1749-0 (PMC4521363; doi:10.1186/s12864-015-1749-0)
Supplement: Additional file 2: Table S1. — Summary statistics of reference and variant allele read counts. The total number of SNPs is 308,912. [file 12864_2015_1749_MOESM2_ESM.doc]

**Additional file 2: Table S1 Summary statistics of reference and variant allele read counts.** The total number of SNPs is 308,912.

|  | **Min** | **1st Qu.** | **Median** | **3rd Qu.** | **Max** | **Mean** | **Variance** |
| --- | --- | --- | --- | --- | --- | --- | --- |
| **raw_ref** | 3 | 4 | 6 | 11 | 4667 | 11.772 | 1174.653 |
| **library_size_adjusted_ref** | 1 | 3 | 5 | 9 | 2805 | 8.806 | 595.878 |
| **raw_var** | 3 | 4 | 6 | 11 | 3128 | 11.025 | 924.083 |
| **library_size_adjusted_var** | 1 | 2 | 4 | 8 | 2413 | 8.271 | 507.409 |
