# Supplementary material for: Analyzing allele specific RNA expression using mixture models
Source: BMC Genomics. 2015 Aug 1;16(1):566. doi: 10.1186/s12864-015-1749-0 (PMC4521363; doi:10.1186/s12864-015-1749-0)
Supplement: Additional file 5: Table S2. — SNPs classified in folded Skellam mixture component Mix3 and Mix5. “ref” and “var” are the original read counts of reference and variant alleles without the adjustment for library sizes. Abs.Ratio = Max(ref, var) / Min(ref, var). “Abs.Adj.Dif” is the absolute value of read difference between reference and variant alleles after library size adjustments. {Pi}, i = 1, 2, … 6, are the mixture probabilities corresponding to each of the six folded Skellam mixture components. Only SNPs in 3’ UTR were used for fitting folded Skellam mixture. [file 12864_2015_1749_MOESM5_ESM.doc]

**Additional file 5: Table S2 SNPs classified in folded Skellam mixture component Mix3 and Mix5.** “ref” and “var” are the original read counts of reference and variant alleles without the adjustment for library sizes. Abs.Ratio= Max(ref, var) / Min(ref, var). “Abs.Adj.Dif” is the absolute value of read difference between reference and variant alleles after library size adjustments.
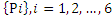
, are the mixture probabilities corresponding to each of the six folded Skellam mixture components. Only SNPs in 3’ UTR were used for fitting folded Skellam mixture.

| **SNP** | **ref** | **var** | **Abs.Ratio** | **Abs.Adj.Dif** | **P1** | **P2** | **P3** | **P4** | **P5** | **P6** |
| --- | --- | --- | --- | --- | --- | --- | --- | --- | --- | --- |
| rs73414847 | 306 | 79 | **3.873** | 186 | **0** | 0 | 0.9988 | 0 | 0.0012 | **0** |
| rs998754 | 21 | 102 | **4.857** | 129 | **0** | 0 | 0.0825 | 0.2236 | 0.6938 | **0** |
| rs77764633 | 277 | 55 | **5.036** | 133 | **0** | 0 | 0.1652 | 0.1043 | 0.7305 | **0** |
| rs74074295 | 411 | 128 | **3.211** | 170 | **0** | 0 | 0.9861 | 0 | 0.0139 | **0** |
| rs1045450 | 744 | 339 | **2.195** | 221 | **0** | 0 | 1 | 0 | 0 | **0** |
