# Supplementary material for: Analyzing allele specific RNA expression using mixture models
Source: BMC Genomics. 2015 Aug 1;16(1):566. doi: 10.1186/s12864-015-1749-0 (PMC4521363; doi:10.1186/s12864-015-1749-0)
Supplement: Additional file 7: Table S4. — Uncertain Signal SNPs with absolute read ratios greater than or equal to 7. “ref” and “var” are the original read counts of reference and variant alleles without the adjustment for library sizes. Abs.Ratio = Max(ref, var) / Min(ref, var). “Abs.Adj.Dif” is the absolute value of read difference between reference and variant alleles after library size adjustments. {Pi}, i = 1, 2, … 6, are the mixture probabilities corresponding to each of the six folded Skellam mixture components. “Comp.” is the assigned folded Skellam mixture component. [file 12864_2015_1749_MOESM7_ESM.doc]

**Additional file 4: Table S4 Uncertain Signal SNPs with absolute read ratios greater than or equal to 7.** “ref” and “var” are the original read counts of reference and variant alleles without the adjustment for library sizes. Abs.Ratio= Max(ref, var) / Min(ref, var). “Abs.Adj.Dif” is the absolute value of read difference between reference and variant alleles after library size adjustments.
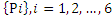
, are the mixture probabilities corresponding to each of the six folded Skellam mixture components. “Comp.” is the assigned folded Skellam mixture component.

| **SNP** | **ref** | **Var** | **Abs.ratio** | **Adj.Abs.Dif** | **P1** | **P2** | **P3** | **P4** | **P5** | **P6** | **Comp.** |
| --- | --- | --- | --- | --- | --- | --- | --- | --- | --- | --- | --- |
| rs115141047 | **30** | **4** | 8 | 24 | 0.622 | 0.377 | 0 | 0.001 | 0 | 0 | 1 |
| rs35674 | **3** | **24** | 8 | 12 | 0.898 | 0.097 | 0 | 0 | 0 | 0.005 | 1 |
| rs1055253 | **36** | **5** | 7 | 24 | 0.622 | 0.377 | 0 | 0.001 | 0 | 0 | 1 |
| rs7566 | **5** | **39** | 8 | 24 | 0.622 | 0.377 | 0 | 0.001 | 0 | 0 | 1 |
| rs9347 | **3** | **27** | 9 | 23 | 0.661 | 0.339 | 0 | 0.001 | 0 | 0 | 1 |
| rs1126878 | **22** | **3** | 7 | 18 | 0.803 | 0.196 | 0 | 0 | 0 | 0 | 1 |
| rs7020 | **3** | **26** | 9 | 27 | 0.512 | 0.486 | 0 | 0.002 | 0 | 0 | 1 |
| rs10898931 | **3** | **29** | 10 | 21 | 0.727 | 0.272 | 0 | 0 | 0 | 0 | 1 |
| rs2016057 | **3** | **23** | 8 | 14 | 0.875 | 0.125 | 0 | 0 | 0 | 0.001 | 1 |
| rs2554315 | **3** | **24** | 8 | 14 | 0.875 | 0.125 | 0 | 0 | 0 | 0.001 | 1 |
