# Supplementary material for: Analyzing allele specific RNA expression using mixture models
Source: BMC Genomics. 2015 Aug 1;16(1):566. doi: 10.1186/s12864-015-1749-0 (PMC4521363; doi:10.1186/s12864-015-1749-0)
Supplement: Additional file 10: Table S7. — SNPs classified as AEI signal SNPs and the corresponding gene classified as eQTLs (n = 18). P-values are calculated using linear regression. [file 12864_2015_1749_MOESM10_ESM.doc]

**Additional file 10: Table S7 SNPs classified as AEI signal SNPs and the corresponding gene classified as eQTLs (n=18).** P-values are calculated using linear regression.

| **Gene** | **rs number** | **P-value** |
| --- | --- | --- |
| PILRB | rs705866 | 6.90E-20 |
| FGF1 | rs34000 | 4.22E-04 |
| SCD | rs3978768 | 2.10E-03 |
| CBX5 | rs4759348 | 0.01 |
| CYFIP2 | rs6862302 | 0.01 |
| NAPB | rs8615 | 0.01 |
| PAFAH1B1 | rs4790356 | 0.02 |
| SLC24A2 | rs7022987 | 0.02 |
| ATP9A | rs1048807 | 0.02 |
| CYFIP2 | rs3734034 | 0.03 |
| BC016978,C1orf61 | rs3431 | 0.03 |
| SLC12A5 | rs9074 | 0.03 |
| USH1C | rs1055577 | 0.04 |
| PDE1B | rs10783631 | 0.04 |
| DDX17 | rs1043402 | 0.04 |
| SLC24A2 | rs7867513 | 0.04 |
| PMP2 | rs6473276 | 0.05 |
| SCD5 | rs1065403 | 0.05 |
